# *Labiobaetis* from the Kingdom of Saudi Arabia (Insecta, Ephemeroptera, Baetidae)

**DOI:** 10.3897/zookeys.774.25273

**Published:** 2018-07-16

**Authors:** Jean-Luc Gattolliat, Boris C. Kondratieff, Thomas Kaltenbach, Hathal M. Al Dhafer

**Affiliations:** 1 Museum of Zoology, Palais de Rumine, Place de la Riponne 6, 1014 Lausanne, Switzerland; 2 Department of Ecology and Evolution, Biophore, University of Lausanne, 1015 Lausanne, Switzerland; 3 Department of Bioagricultural Sciences and Pest Management, Colorado State University, Fort Collins, CO 80523, USA; 4 Plant Protection Department, College of Food and Agriculture Sciences, King Saud University, Riyadh 11451, P. O. Box 2460, Saudi Arabia

**Keywords:** Arabian Peninsula, mayflies, new species, new records

## Abstract

Mayfly larvae and imagos were collected at approximately 50 localities of the Kingdom of Saudi Arabia (KSU). Included in this material, three species of *Labiobaetis* Novikova & Kluge, 1987 are recorded, two of them being new to science. *Labiobaetis potamoticus* Gattolliat & Al Dhafer, **sp. n.** is described from both larvae and adults, whereas *Labiobaetis alahmadii* Gattolliat & Al Dhafer, **sp. n.** is only known from the larval stage. The two species are compared morphologically with Palearctic and Afrotropical species of *Labiobaetis*. A third species, *Labiobaetis glaucus* (Agnew, 1961) is reported for the first time from the Arabian Peninsula. The species was originally described from South Africa and subsequently reported from the east and northeast of Africa. A molecular reconstruction including 18 Afrotropical and Palearctic species of *Labiobaetis* was performed using 658 bp of the mitochondrial gene CO1. The reconstruction highly supported the validity of the two new species and confirmed the occurrence of *L. glaucus* in KSU.

## Introduction

*Labiobaetis* Novikova & Kluge, 1987 is a species-rich genus with an almost worldwide distribution (only absent in Neotropical Region); it is mainly diversified in Afrotropical (28 species) and Oriental realms (23 species) (Gattolliat and Nieto 2009). The status and validity of the genus has often been the subject of controversy during the last two decades (Fujitani 2008; Fujitani et al. 2003; Gattolliat 2001; Gattolliat and Staniczek 2011; Kluge 2012; Kluge and Novikova 2016; Kubendran et al. 2015; Lugo-Ortiz and McCafferty 1997; Lugo-Ortiz et al. 1999). Molecular reconstructions indicated that the concept of *Labiobaetis* is most probably at least diphyletic (Gattolliat et al. 2008; Monaghan et al. 2005). Larvae of *Labiobaetis* generally colonize the more lentic portion of streams and rivers with rich aquatic vegetation where it can be the most abundant mayfly.

The distribution of the genus in Arabian Peninsula seems restricted to the southwestern Kingdom of Saudi Arabia (KSU) and Yemen despite potential suitable habitats present in Oman, Jordan and the United Arab Emirates (Gattolliat and Sartori 2008; Gattolliat et al. 2012).

Relatively little information is available on the freshwater habitats and faunas of the Arabian Peninsula and, in particular, of the KSU. The vast landscape of KSU is one of the driest and hottest countries in the world and has almost no runoff, surface water or perennial rivers (Alshareef 1995, Alkolibi 2002, Al-Rashed and Sherif 2000). Other than the Midwestern Region and the southwestern mountains, the average annual precipitation in KSU ranges from 80 mm to 140 mm and maximum summer temperatures often exceeds 45 °C (Alkolibi 2002). Seasonal rainfall occurs more frequently and in greater quantity in the southwestern Hejaz and Asir mountains, therefore most permanent and semi-permanent lotic drainages or wadis occur in this region of KSU (Crosskey and Büttiker 1982, Alkahem and Behnke 1983, Whitton et al. 1986, Sorman and Abdulrazzak 1993, Al-Ghamdi and Abu-Zinadah 1998). These wadis are usually the only habitats for mayflies. Many of these drainages however, have been dammed to capture surface water. More than 230 dams now store annual runoff in reservoirs (Kingdom of Saudi Arabia Ministry of Foreign Affairs 2015). This water is used primarily for agriculture and is distributed through thousands of kilometers of irrigation canals, which can also support mayfly populations. Habitats such as oases (Edgell 2006) in KSU have not been adequately sampled for mayflies.

The first systematic studies of the mayflies of KSU were published more than 25 years ago. Six species belonging to three families (Baetidae, Caenidae and Leptophlebiidae) were mentioned from KSU (Sartori 1991; Sartori and Gillies 1990; Thomas and Sartori 1989). Within Baetidae, *Baetis balcanicus* Müller-Liebenau & Soldán, 1981, *Centroptilum dimorphicum* Soldán & Thomas, 1985 and *Cloeon saharense* Soldán & Thomas, 1983 were reported (Sartori 1991; Sartori and Gillies 1990; Thomas and Sartori 1989). *Baetis balcanicus* is now considered as a *Labiobaetis* (Lugo-Ortiz and McCafferty 1997; McCafferty and Waltz 1995). The validity of this identification is debated below in the discussion section. *Centroptilum dimorphicum* is now assigned to the genus *Cheleocloeon* Wuillot & Gillies, 1993 (Wuillot and Gillies 1993). The attribution of the specimens collected in the Arabian Peninsula to a species previously known only from North-West Africa appears now highly questionable, especially as a new species of *Cheleocloeon*, *C. soldani* Gattolliat & Sartori, 2008, was described from United Arab Emirates (Gattolliat and Sartori 2008). We therefore consider the above specimens of *Centroptilum dimorphicum* reported from KSU as belonging to Cheleocloeon cf. soldani. Close morphological analysis is required before a definitive species assignment can be decided. At least two species of *Cloeon* Leach, 1815 occur in KSU and they appear both morphologically and genetically more related to Afrotropical species of *Cloeon* rather than to North African species such as *Cloeon saharense* (Salles et al. 2014).

## Material and methods

The majority of the material was collected in November 2012 during a scientific expedition organised by King Saud University Museum of Arthropods, College of Food and Agriculture Sciences, Department of Plant Protection, King Saud University, KSU. Mayflies were collected from approximately 50 localities mainly along the southern coast of KSU. Other specimens were collected during different fieldtrips led by the same institution mainly in 2010 and 2012. Larvae were collected primarily by kick netting in stream vegetation along edges of wadis. Imagos were collected by light traps using black light with a white sheet. Mature larvae were reared in rearing chambers; larval and subimaginal exuviae were collected and stored with the corresponding imago. All material is stored in 80% ethanol. Holotypes and part of the paratypes are deposited in the King Saud University Museum of Arthropods, Riyadh, Saudi Arabia (KSU); other paratypes are housed in the Museum of Zoology, Lausanne, Switzerland (MZL). Each GBIFCH code refers to a tube with group of specimens in or a slide with a single specimen (sequenced or not).

The association of the ontogenetic stages was easily made for reared material. For specimens obtained by light trapping and kick sampling, we used sequence divergence of the fragment of mitochondrial cytochrome c oxidase subunit I (CO1) gene. Specimens belonging to the different “morphospecies” and collected in the same localities were selected for genetic analysis. The CO1 gene was sequenced using LCO1490 and HCO2198 primers (Folmer et al. 1994). We followed the laboratory procedures, edition and alignment of sequences as described in Vuataz et al. (2011). The final data matrix included 41 CO1 sequences of 658 bp representing all the *Labiobaetis* taxa from Palearctic and Afrotropical regions for which sequences are available (Table 1). Analyses were conducted in MEGA7 (Kumar et al. 2016).

**Table 1.** Specimens used for the phylogenetic analysis of the mitochondrial gene CO1.

Species	Locality	Specimen catalog #	GenBank # (COI)	GenSeq Nomenclature
*Labiobaetis glaucus*	South Africa	GBIFCH00517537	MH070310	genseq-4 COI
	South Africa	GBIFCH00517539	MH070321	genseq-4 COI
	South Africa	GBIFCH00517538	MH070319	genseq-4 COI
	Mayotte	GBIFCH00517531	MH105069	genseq-4 COI
	Mayotte	GBIFCH00521580	MH070315	genseq-4 COI
	Mayotte	GBIFCH00517530	MH070318	genseq-4 COI
	Saudi Arabia	GBIFCH00465151	MH070288	genseq-4 COI
	Saudi Arabia	GBIFCH00235741	MH070311	genseq-4 COI
	Saudi Arabia	GBIFCH00235750	MH105068	genseq-4 COI
	Saudi Arabia	GBIFCH00235731	MH070317	genseq-4 COI
	Saudi Arabia	GBIFCH00517523	MH070320	genseq-4 COI
*Labiobaetis potamoticus* sp. n.	Saudi Arabia	GBIFCH00517520	MH070306	genseq-2 COI
	Saudi Arabia	GBIFCH00517521	MH070308	genseq-2 COI
	Saudi Arabia	GBIFCH00235735	MH070312	genseq-2 COI
	Saudi Arabia	GBIFCH00235732	MH070316	genseq-2 COI
	Saudi Arabia	GBIFCH00465152	MH070289	genseq-2 COI
	Saudi Arabia	GBIFCH00465154	MH070290	genseq-2 COI
	Saudi Arabia	GBIFCH00517527	MH070307	genseq-2 COI
	Saudi Arabia	GBIFCH00235747	MH070313	genseq-2 COI
	Saudi Arabia	GBIFCH00235757	MH070314	genseq-2 COI
	Saudi Arabia	GBIFCH00517526	MH070322	genseq-2 COI
	Saudi Arabia	GBIFCH00465155	MH070291	genseq-2 COI
*Labiobaetis boussoulius*	Ivory Coast	GBIFCH00517528	MH070309	genseq-4 COI
*Labiobaetis* sp.	Ivory Coast	GBIFCH00465136	MH070294	
*Labiobaetis* sp.	Ivory Coast	GBIFCH00465137	MH070295	
*Labiobaetis* sp.	Ivory Coast	GBIFCH00465138	MH070296	
*Labiobaetis* sp.	South Africa	GBIFCH00465153	MH070305	
*Labiobaetis* sp.	South Africa	GBIFCH00465135	MH070303	
*Labiobaetis piscis*	South Africa		IBOL CED 150U	genseq-4 COI
*Labiobaetis latus*	South Africa	GBIFCH00465142	MH070297	genseq-4 COI
*Labiobaetis vinosus*	South Africa	GBIFCH00465143	MH070304	genseq-4 COI
*Labiobaetis dambrensis*	Madagascar	GBIFCH00465144	MH070293	genseq-2 COI
*Labiobaetis nigrocercus*	Madagascar	GBIFCH00465145	MH070300	genseq-4 COI
*Labiobaetis longicercus*	Madagascar	GBIFCH00465146	MH070298	genseq-4 COI
	Madagascar	GBIFCH00465147	MH070299	genseq-4 COI
*Labiobaetis punctatus*	Madagascar	GBIFCH00465148	MH070301	genseq-4 COI
	Madagascar	GBIFCH00465149	MH070302	genseq-4 COI
*Labiobaetis atrebatinus*	France	GBIFCH00465150	MH070292	genseq-4 COI
*L. atrebatinus orientalis*	Japan		KF563032	genseq-4 COI
*Labiobaetis tricolor*	Hungary		JN164313	genseq-4 COI
*Baetis rhodani*	Switzerland		HG935037	genseq-4 COI

Tree topology was reconstructed using the Maximum Likelihood method based on the Tamura-Nei model (Tamura and Nei 1993). The tree with the highest log likelihood is shown. The percentage of trees in which the associated taxa clustered together is shown next to the branches (Bootstrap with 1000 replicates). We used 3.5% sequence divergence (measured by Kimura 2-parameter (K2P)) as the maximal value for intraspecific divergence (Ball et al. 2005; Gattolliat et al. 2015; Rutschmann et al. 2014; Rutschmann et al. 2017; Webb et al. 2012). As part of the sequenced specimens came from the same population, the intraspecific divergence is expected to be even clearly lower than the limit.

## Taxonomy

### Labiobaetis potamoticus

Taxon classificationAnimaliaEphemeropteraBaetidae

Gattolliat & Al Dhafer sp. n.

http://zoobank.org/EAAD82B8-5680-49C0-879C-8B4F204FF351

Figs 1–8 , 9–15 , 16–19

Baetis balcanicus Müller-Liebenau & Soldán, 1981 in Thomas and Sartori 1989: 87.

#### Type material.

**Holotype**: Male larva (GBIFCH00521578): Saudi Arabia (AR44); Wadi Shahadan; 17°28'36"/ 42°42'50"; Alt. 190m; 13.XI.2012; Coll. J-L Gattolliat.

**Paratypes**: 4 larvae (GBIFCH00235716 + GBIFCH00235735 (Genetics)), 1 male imago (GBIFCH00235732 (Genetics)) + 3 larvae (KSU: GBIFCH00526192); same data as holotype.

42 larvae (GBIFCH00235729 + 3 slides GBIFCH00235758, GBIFCH00235733, GBIFCH00235760): Saudi Arabia (AR01); Al Jiwah, Thee Aine; 19°55'32"/ 41°26'17"; Alt. 752m; 13.X.2010; Coll. B. Kondratieff.

14 larvae (GBIFCH00235721): Saudi Arabia (AR20); Wadi Baqrah; 18°47'29"/ 41°56'19"; Alt. 490m; 13.III.2012; Coll. Al Dhafer, H. & Kondratieff, B.

5 larvae (GBIFCH00235722): Saudi Arabia (AR28); Thee Ain, Al-Baha; 19°55'46"/ 41°26'34"; Alt. 760m; 3.VI.2012; Coll. Al Dhafer, H. & Kondratieff, B.

2 larvae (GBIFCH00235714): Saudi Arabia (AR31); Thee Ain, Al-Baha; 19°55'46"/ 41°26'34"; Alt. 760m; 8.XI.2012; Coll. J-L Gattolliat.

58 larvae (GBIFCH00235706, GBIFCH00235728, GBIFCH00235717 + 2 slides GBIFCH00235749, GBIFCH00235759 + GBIFCH00517520 (Genetics), GBIFCH00517521 (Genetics)) + 11 larvae (KSU: GBIFCH00526173): Saudi Arabia (AR32); Wadi Elarj, near Adam; 20°27'11"/ 40°48'56"; Alt. 440m; 9.XI.2012; Coll. J-L Gattolliat.

23 larvae (GBIFCH00235726, GBIFCH00235719 + 3 slides GBIFCH00235753, GBIFCH00465152 (Genetics), GBIFCH00465155 (Genetics)) + 4 larvae (KSU: GBIFCH00526224): Saudi Arabia (AR43a); Wadi Shahadan; 17°28'36"/ 42°51'25"; Alt. 460m; 12.XI.2012; Coll. J-L Gattolliat.

3 larvae (KSU: GBIFCH00526237): Saudi Arabia (AR43b); Wadi Shahadan; 17°28'17"/ 42°51'14"; Alt. 455m; 12.XI.2012; Coll. J-L Gattolliat.

#### Additional material.

1 larva (on slide), Saudi Arabia, Wadi Buwah, 1340m, 20°47'N / 41°12'E, 20.IX.1980, Leg. W. Büttiker. Coll. Naturhistorisches Museum Basel, Switzerland. (Previously identified as *Baetis balcanicus*, det. A. Thomas).

#### Differential diagnosis.

Larva: Tergites I-X medium brown with peculiar pattern formed of six ecru dots (Fig. 19). Scape of antenna without distolateral process (Fig. 13). Segment II of the maxillary palp without a distomedial concavity (Fig. 7). Segment II of labial palp with a slender triangular distomedial projection (Fig. 8). Dorsal margin of femur with a few medium setae, not abundant proximally; ventral margin with abundant medium setae (Fig. 10). Dorsal margin of tibia almost bare (Fig. 9). Paraproct with numerous distal spines (Fig. 15). Male imago: Genitalia with inner margin of segments I and II without expansion; segment III almost globular (Fig. 18).

#### Description.

**Larva.** *Length*: fully grown female: Body 5.1–7.7 mm, cerci 3.6–4.0 mm, terminal filament 2.5–2.6 mm. Fully grown male: Body 5.0–5.3 mm, cerci 3.4–3.6 mm, terminal filament 2.5 mm.

*Colouration* (Fig. 19): Head almost uniformly brown with ecru vermiform marking on vertex and frons, border of sclerites yellow. Prothorax medium brown, lighter laterally; mesothorax medium brown with a central yellow dot and a transversal yellow stripe; metathorax medium brown. Legs: ecru except a brown central dot on femora; dorsal and ventral margin of femora brown, dorsal margin of tibiae and tarsi brown. Tergites I-X medium brown with peculiar pattern formed of six ecru dots sometimes fused. Abdominal sternites brown sometimes with four ecru dots. Cerci ecru brown getting progressively ecru towards apex.

*Head*: scape of antenna without distolateral process (Fig. 13).

**Figures 1–8.**
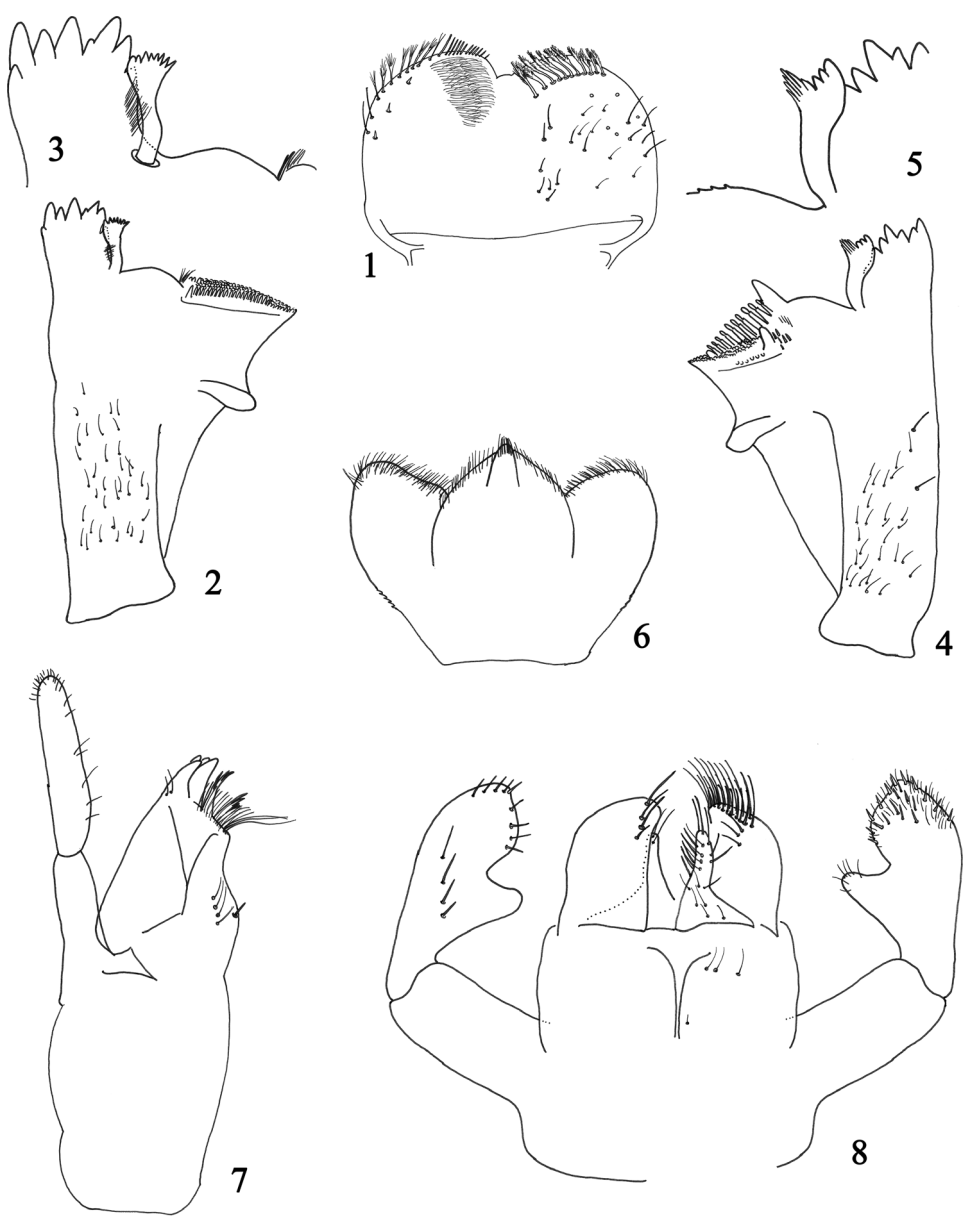
Larval structures of *Labiobaetis potamoticus* sp. n.: **1** labrum (left : ventral; right : dorsal) **2** right mandible **3** detail of right mandible: canines and prostheca **4** left mandible **5** detail of left mandible: prostheca **6** hypopharynx **7** right maxilla **8** labium.

*Labrum* (Fig. 1) rounded with a small anteromedial emargination, dorsally with a distolateral row of approx. eight feathered setae, without a submedian seta; short, thin, simple setae scattered on dorsal surface of labrum; distal margin bordered with feathered setae.

*Right mandible* (Fig. 2): canine with two almost fused incisivi each with four denticles, outer denticle much shorter than others, inner margin of inner incisive with a row of very thin setae; stout prostheca apically with small pointed denticles (Fig. 3); margin between prostheca and mola slightly convex, smooth, without setae; tuft of setae at apex of mola absent.

*Left mandible* (Fig. 4): canine with two almost fused incisivi each with four denticles, outer denticle much shorter than others; stout prostheca apically with small denticles and a comb-shaped structure (Fig. 5); margin between prostheca and mola slightly concave, without crenulations; tuft of setae at apex of mola absent.

*Hypopharynx* as illustrated in Fig. 6.

*Maxilla* (Fig. 7) with a medioapical row of medium setae, basal end of row with a few long setae; posterior side of lacinia mediobasally with a row of four medium-sized setae, a single small seta close to the medial margin of lacinia; palp 2-segmented, longer than galea-lacinia, segment II without distomedial concavity.

*Labium* (Fig. 8) with glossae shorter than paraglossae; glossae inner margin with two rows of approx. six long setae, apically with a few simple setae; paraglossae stout, apically flattened, with three rows of long simple setae; labial palp with segment I slender, quadrangular, shorter than segments II and III combined; segment II with a slender, elongated distomedial projection with few thin setae apically, on posterior side with a row of four long setae increasing in length; segment III subconical, inner margin apically slightly concave, with abundant scattered short thin setae and stouter setae.

*Thorax*: hind wing pads present.

**Figures 9–15.**
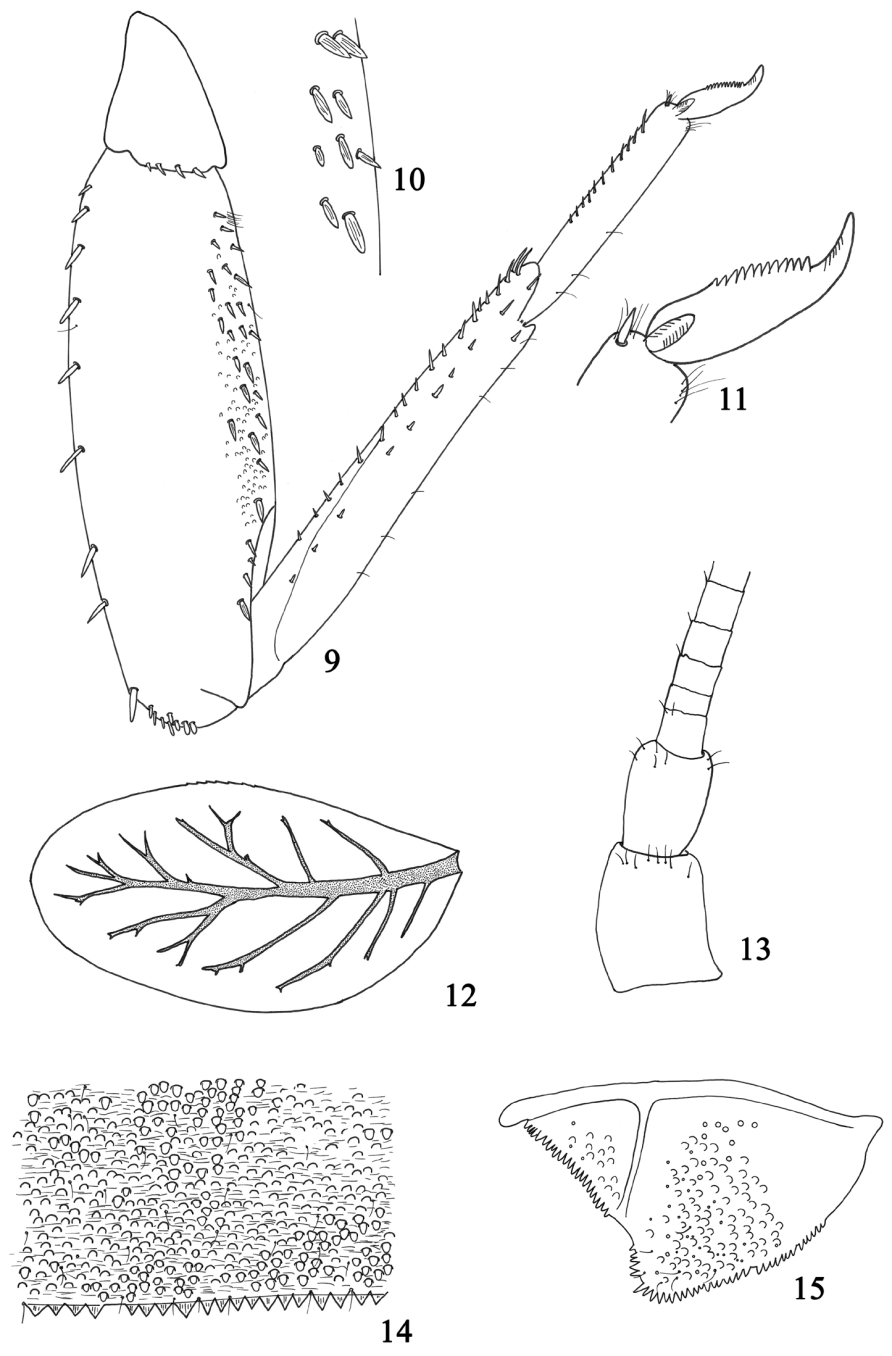
Larval structures of *Labiobaetis potamoticus* sp. n.: **9** foreleg **10** detail of ventral margin of forefemora **11** tarsal claw **12** fourth gill **13** base of antenna **14** posterior margin of fourth abdominal tergite **15** paraproct.

*Legs* (Fig. 9): forefemur dorsally with a row of approx. eight medium-sized, apically rounded setae; apex with one medium stout seta and several short flattened setae; ventral margin with a poorly developed villopore and abundant, medium, stout setae (Fig. 10). Foretibia dorsally almost bare; ventrally with short setae, only slightly longer apically. Foretarsus almost bare dorsally; ventral margin with a row of pointed setae slightly increasing in length toward apex; tarsal claw (Fig. 11) with a single row of approx. twelve pointed teeth; without subapical setae. Middle and hind legs similar to foreleg but with reduced setation.

*Abdomen*: tergites (Fig. 14) shagreened with numerous scales and scale bases, with a few setae; posterior margin with triangular spination as long as broad. Sternites with scales and scale bases; posterior margin smooth, without spines.

*Gills* present on abdominal segments I–VII (Fig. 12), poorly serrated, tracheation brown, with abundant ramifications.

*Paraproct* (Fig. 15) with scale bases and a few setae, margin with numerous small triangular spines regular in size; posterolateral extension with a few scale bases, spines along the margin of the same size as those of the paraproct.

**Male imago**

*Length*. Body 4.8 mm; forewing 4.4 mm; hindwing 0.8 mm.

**Figures 16–19.**
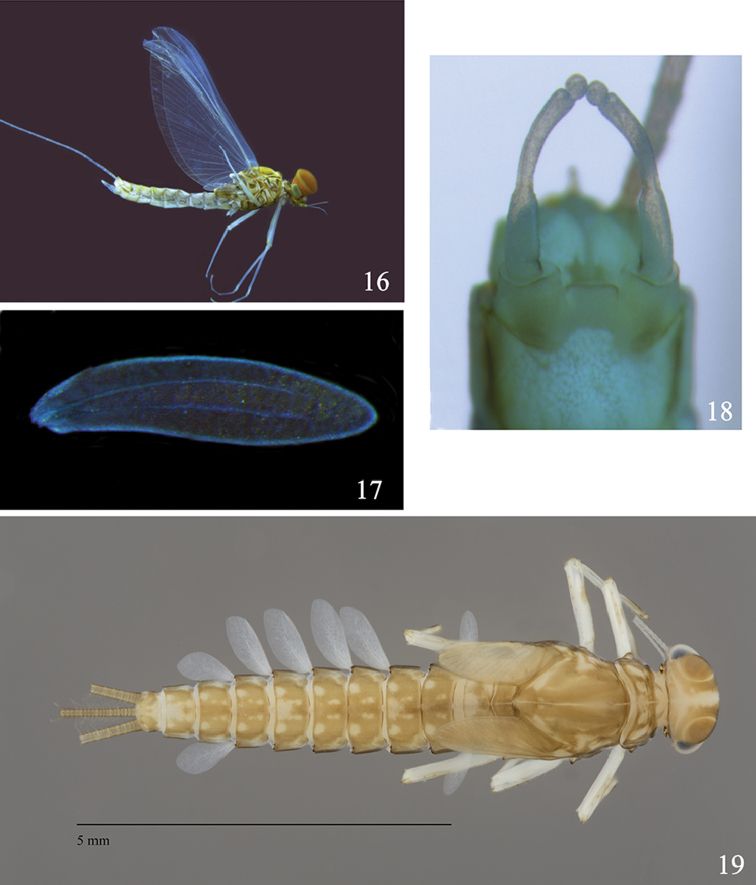
Larval and imaginal structures of *Labiobaetis potamoticus* sp. n.: **16** male imago (lateral view) **17** hindwing **18** male genitalia **19** male larva (dorsal view).

*Colouration*: head brown; antenna ecru except base of scape and pedicel brown. Facetted surface of turbinate eyes dark orange brown, shaft orange brown, lighter apically (Fig. 16). Thorax yellowish brown with margin of sclerites generally dark brown. Legs: yellowish without marks or pattern. Wings hyaline, hyaline venation. Abdomen: tergites I to X ecru without pattern. Sternites I to IX ecru. Cerci ecru. Genitalia (Fig. 18) ecru except inner margin of segment I medium brown.

Forewing: pterostigma with approx. two cross-veins not reaching subcostal vein; double intercalary veins shorter than distance between corresponding main veins.

Hindwing (Fig. 17) without costal spur; two longitudinal veins almost reaching margin, none of them bifurcated, without incomplete veinlets between main longitudinal veins.

Genitalia (Fig. 18): basal segment with inner margin not expanded apically; segments I and II almost completely fused; constriction at basis of segment II; segment III globular.

#### Distribution and habitat.

*Labiobaetis potamoticus* appears to be the most widespread species of *Labiobaetis* in KSU. It colonizes aquatic vegetation in still reaches of small to medium-sized streams with a sandy substrate (Figs 48, 52). This species was also collected in a secondary channel with *Typha* sp. (Fig. 49). This species seems less rheophilic than the two following taxa.

#### Etymology.

The specific name *potamic*- was given in reference to the ecological preferences of the species for still and standing water.

### Labiobaetis alahmadii

Taxon classificationAnimaliaEphemeropteraBaetidae

Gattolliat & Al Dhafer sp. n.

http://zoobank.org/605BFF9A-A54E-419F-912B-462B1E9FAAF9

#### Type material.

**Holotype**: Female larva (GBIFCH00521579): Saudi Arabia (AR43a); Wadi Shahadan; 17°28'36"/ 42°51'25"; Alt. 460m; 12.XI.2012; Coll. J-L Gattolliat.

**Paratypes**: 151 larvae (GBIFCH00235715, GBIFCH00235720 + GBIFCH00517525 + 3 slides GBIFCH00235737, GBIFCH00235755, GBIFCH00235757 (Genetics), GBIFCH00235737) + 8 larvae (KSU: GBIFCH00526208): same data as holotype.

47 larvae (GBIFCH00235710) + 10 larvae (KSU: GBIFCH00526227): Saudi Arabia (AR43b); Wadi Shahadan; 17°28'17"/ 42°51'14"; Alt. 455m; 12.XI.2012; Coll. J-L Gattolliat.

29 larvae (GBIFCH00235727) + 6 larvae (KSU: GBIFCH00526223): Saudi Arabia (AR39); Wadi Damad; 17°12'21"/ 43°01'35"; Alt. 260m; 11.XI.2012; Coll. J-L Gattolliat.

15 larvae (GBIFCH00235709 + 3 slides GBIFCH00235744, GBIFCH00235747 (Genetics), GBIFCH00465155 (Genetics) + GBIFCH00517526 (Genetics), GBIFCH00517527 (Genetics)) + 4 larvae (KSU: GBIFCH00526179): Saudi Arabia (AR44); Wadi Shahadan; 17°28'36"/ 42°42'50"; Alt. 190m; 13.XI.2012; Coll. J-L Gattolliat.

#### Differential diagnosis.

**Larva**: *Colouration*: mesothorax medium brown with a W-shaped yellow pattern; tergites I-VIII medium brown with two broad ecru spots, tergites IX and X yellow (Figs 32, 33). Scape of antenna without distolateral process (Fig. 28). Segment II of the maxillary palp with a small distomedial concavity (Fig. 23). Segment II of labial palp with a broad apically rounded triangular distomedial projection (Fig. 24). Dorsal margin of femur with regularly spaced setae; ventral margin almost bare (Fig. 25). Dorsal margin of tibia with a row of small spatulate setae (Fig. 26). Paraproct with approximately eight stout, pointed spines increasing in length toward the apex (Fig. 31).

#### Description.

**Larva.** *Length*: fully grown female: Body 9.5–10.6 mm, cerci 4.0–4.2 mm, terminal filament 2.9–3.1 mm. Fully grown male: Body 8.8–9.9 mm, cerci 3.8–4.1 mm, terminal filament 2.8–2.9 mm.

*Colouration* (Figs 32, 33): head almost uniformly medium brown, with darker, vermiform marking on vertex and frons, border of sclerites yellow. Prothorax ecru with proximal margin medium brown and a brown dot medio-apically; mesothorax medium brown with a double V-shaped yellow pattern; metathorax medium brown. Legs: ecru except femora with a central brown spot and apex of femora, tibiae and tarsi brown. Tergites I-VIII medium brown with two broad ecru spots sometimes fused medially, tergites IX and X yellow. Abdominal sternites ecru getting darker and brownish after sternite VI. Cerci ecru without dark stripe.

*Head*: scape of antenna without distolateral process (Fig. 28).

**Figures 20–24.**
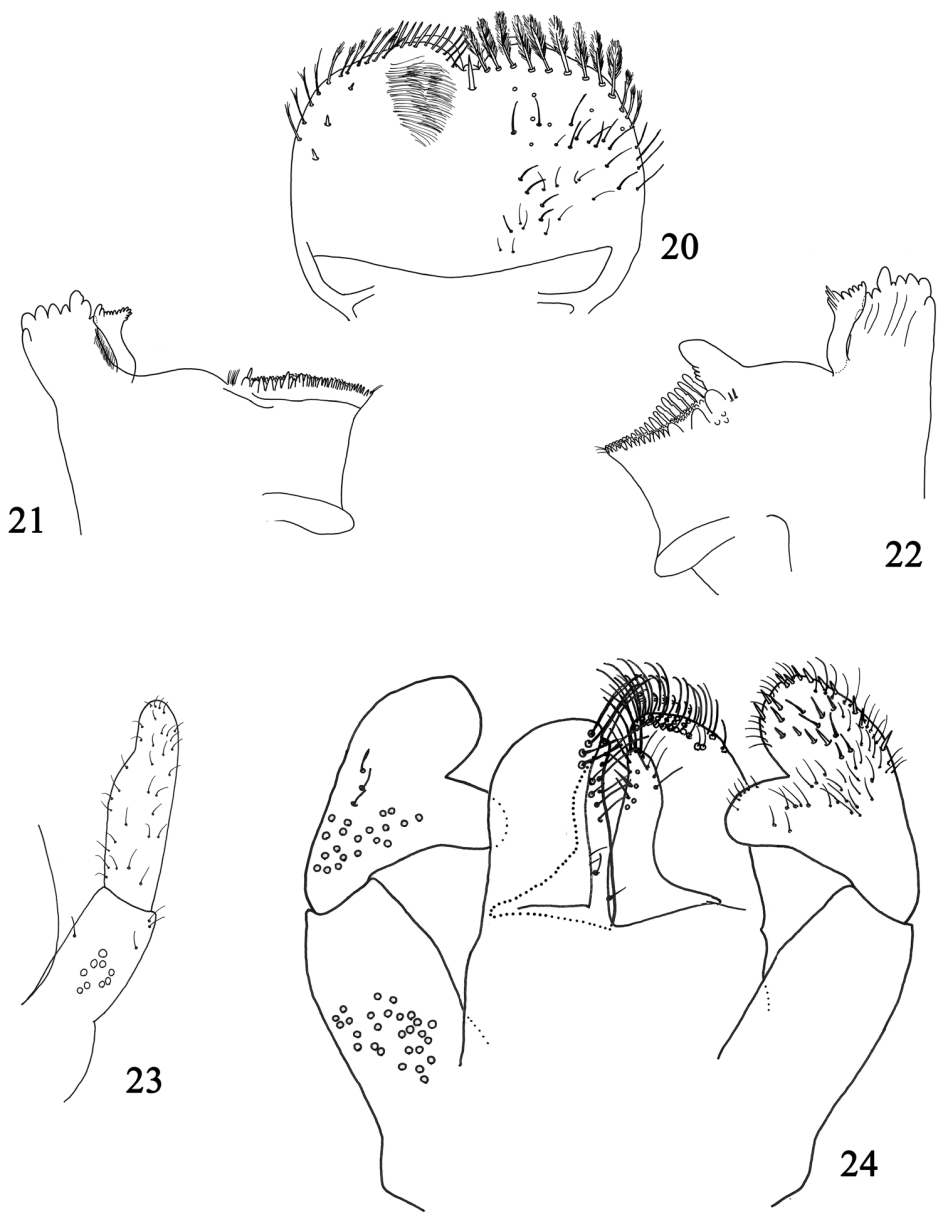
Larval structures of *Labiobaetis alahmadii* sp. n.: **20** labrum (left : ventral; right: dorsal) **21** right mandible **22** left mandible **23** detail of maxilla **24** labium.

*Labrum* (Fig. 20) rounded, with a small anteromedial emargination, dorsally with a relatively short submedian seta and a distolateral row of approx. ten feathered setae; short, thin, simple setae scattered on dorsal surface of labrum; distal margin bordered with feathered setae.

*Right mandible* (Fig. 21): canine with two almost fused incisivi each with four denticles, outer denticle much shorter than others, inner margin of inner incisive with a row of very thin setae; stout prostheca apically with numerous small rounded denticles, also covering distoapical corner; margin between prostheca and mola slightly convex, smooth, without setae; tuft of setae at apex of mola present.

*Left mandible* (Fig. 22): canine with two almost fused incisivi each with four denticles, outer denticle much shorter than others; stout prostheca apically with small denticles and a comb-shaped structure; margin between prostheca and mandible slightly concave, without crenulations; tuft of setae at apex of mola present.

*Hypopharynx* similar to Fig. 6.

*Maxilla* with a medioapical row of medium setae, basal end of row with a few long setae; posterior side of lacinia mediobasally with a row of four medium-sized setae, a single small seta close to the medial margin of lacinia; palp 2-segmented, as long as galea-lacinia, segment II with distomedial concavity (Fig. 23).

*Labium* (Fig. 24) with glossae shorter than paraglossae; glossae with medium to long simple setae in apical half; paraglossae stout, apically rounded, with three rows of setae: two rows with simple setae and one row with setae feathered on one side; labial palp with segment I slender, quadrangular, as long as segments II and III combined; segment II with an elongated triangular distomedial projection with few scattered thin setae, on posterior side with a row of three medium setae; segment III subconical, with abundant scattered short thin setae and stouter setae.

*Thorax*: hind wing pads present.

**Figures 25–31.**
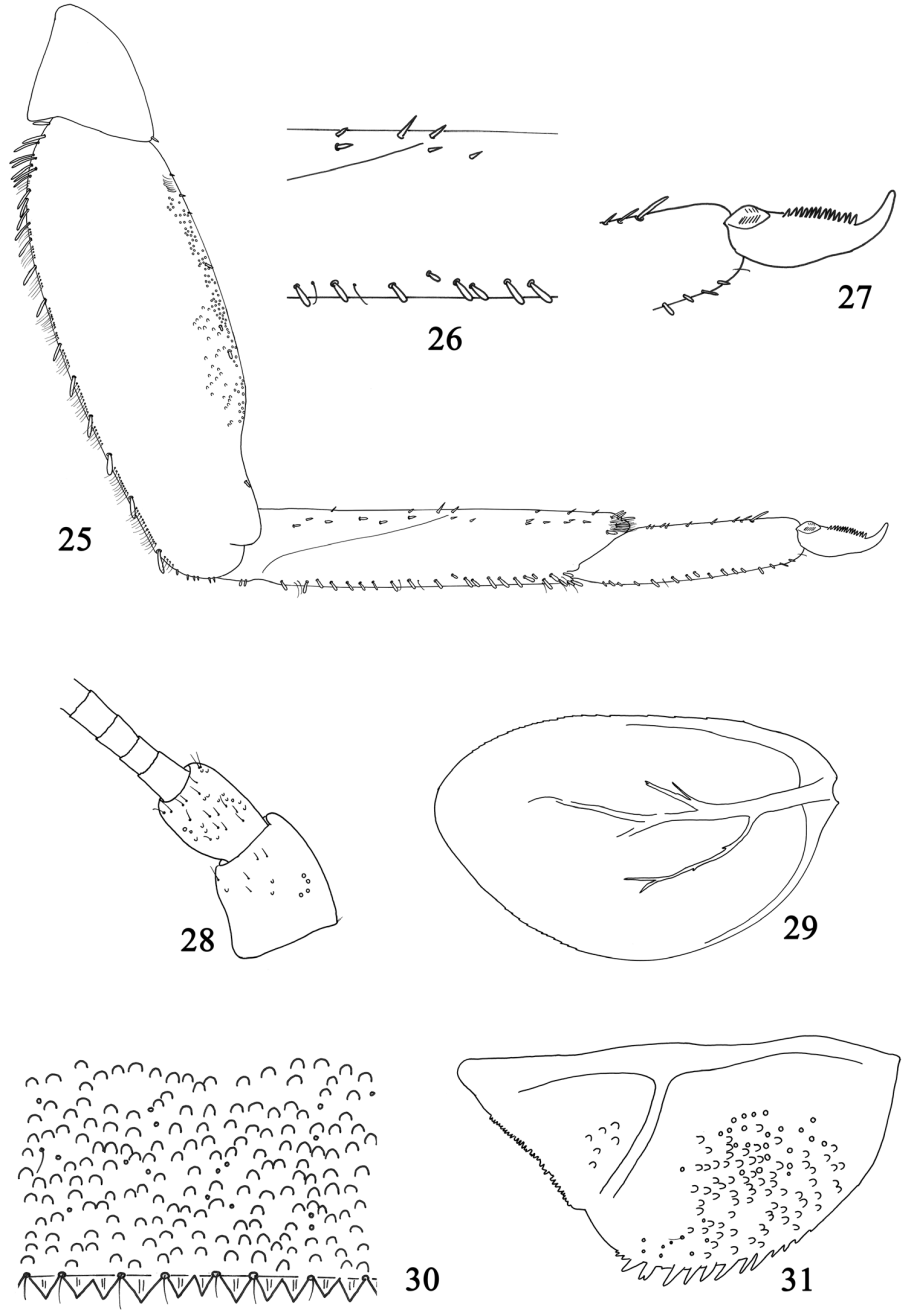
Larval structures of *Labiobaetis alahmadii* sp. n.: **25** foreleg **26** detail of ventral margin of foretibia **27** tarsal claw **28** base of antenna **29** fourth gill **30** posterior margin of fourth abdominal tergite **31** paraproct.

*Legs* (Fig. 25): forefemur dorsally with a row of medium-sized, apically rounded setae, abundant proximally; apex with one short stout seta and minute setae; ventral margin with a poorly developed villopore and scarce minute setae. Foretibia dorsally with a row of short spatulate setae (Fig. 26); ventrally with short setae, not longer apically, apex with a patch of numerous flattened short setae. Foretarsus dorsally with a row of short spatulate setae; ventral margin with a row of minute pointed setae slightly increasing in length toward apex; tarsal claw (Fig. 27) with a single row of approx. 14 pointed teeth; without subapical setae. Middle and hind legs similar to foreleg but with reduced setation.

*Abdomen*: tergites (Fig. 30) with numerous scale bases with a few setae; posterior margin with triangular spination as long as broad. Sternites with numerous scales and scale bases; sternites I-VII with posterior margin smooth without spines, sternites VIII and IX with small triangular spines.

*Gills* present on abdominal segments I–VII, margins serrated, tracheation poorly marked and poorly divided (Fig. 29).

*Paraproct* (Fig. 31) with scale bases, almost bare, margin with approx. eight stout, pointed spines and bordered by few small spines; posterolateral extension with a few scale bases, minute spines along the margin.

**Figures 32–33.**
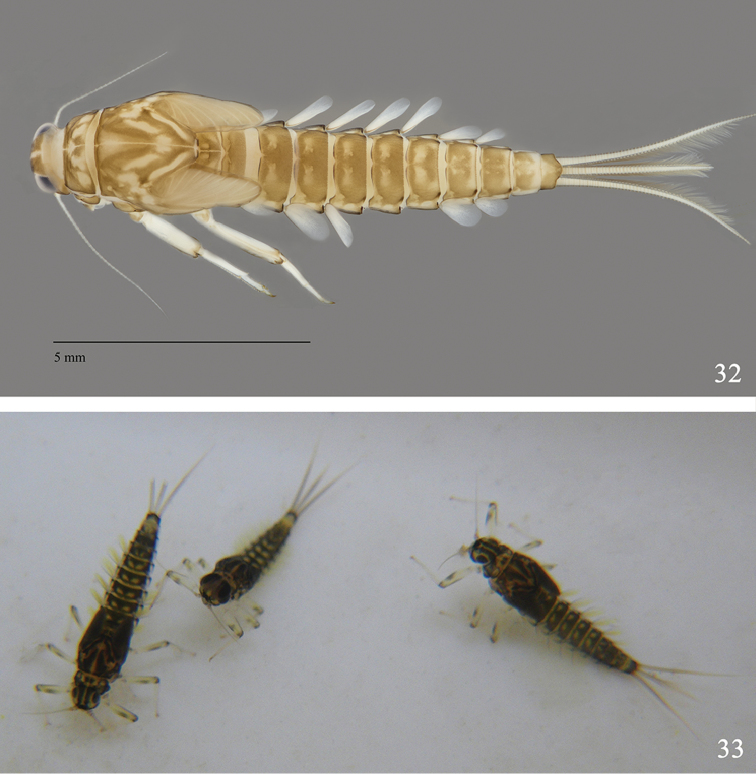
Larvae of *Labiobaetis alahmadii* sp. n.: **32** female larva (dorsal view) **33** larvae in vivo.

**Imagos**: Unknown

#### Distribution and habitat.

We collected this species in only three different localities in close proximity, in extreme southwestern KSU close to the Yemen border. The larvae occur in medium-size streams with stony substrates (Figs 50, 51, 52). Larvae apparently prefer relatively fast flowing reaches. Larvae were even collected at the base of small waterfalls. *Labiobaetis alahmadii* appeared to be an abundant species when the above ecological conditions are optimal. The new species was always sympatric with *L. glaucus*.

#### Etymology.

This species is dedicated to the memory of Professor Ahmed Ziad Al Ahmadi, the well-known Syrian entomologist who passed away few months ago.

### Labiobaetis glaucus

Taxon classificationAnimaliaEphemeropteraBaetidae

(Agnew, 1961)

Figs 34–39 , 40–44 , 45–47

Baetis glaucus Agnew, 1961: 14.
Pseudocloeon glaucum , Lugo-Ortiz et al. 2000: 281.
Labiobaetis glaucus , Kluge and Novikova 2016: 32–33.

#### Specimens examined.

18 larvae (GBIFCH00235711 + 4 slides GBIFCH00235741 (Genetics), GBIFCH00235746, GBIFCH00235750 (Genetics), GBIFCH00235756: Saudi Arabia (AR01); Al Jiwah, Thee Aine; 19°55'32"/ 41°26'17"; Alt. 752m; 13.X.2010; Coll. B. Kondratieff.

3 larvae (GBIFCH00235708): Saudi Arabia (AR19); Wadi Khat; 19°05'22"/ 41°58'16"; Alt. 490m; 13.III.2012; Coll. Al Dhafer, H.

1 larva (GBIFCH00235712): Saudi Arabia (AR28); Thee Ain, Al-Baha; 19°55'43"/ 41°26'34"; Alt. 760m; 3.VI.2012; Coll. Al Dhafer, H. & Kondratieff, B.

3 larvae (GBIFCH00235713): Saudi Arabia (AR31); Thee Ain, Al-Baha; 19°55'43"/ 41°26'34"; Alt. 760m; 8.XI.2012; Coll. J-L Gattolliat.

1 larva (GBIFCH00235707): Saudi Arabia (AR43a); Wadi Shahadan; 17°28'36"/ 42°51'25"; Alt. 460m; 12.XI.2012; Coll. J-L Gattolliat.

2 larvae GBIFCH00465151 (Genetics): Saudi Arabia (AR43b); Wadi Shahadan; 17°28'17"/ 42°51'14"; Alt. 440m; 12.XI.2012; Coll. J-L Gattolliat.

7 larvae (GBIFCH00235723 + 1 slide GBIFCH00235738), 3 male imagos (GBIFCH00235724 + 1 slide GBIFCH00235731 (Genetics)): Saudi Arabia (AR44); Wadi Shahadan; 17°28'36"/ 42°42'50"; Alt. 190m; 13.XI.2012; Coll. J-L Gattolliat.

#### Differential diagnosis.

*Larva*: abdominal pattern (Fig. 47) with tergites I, VI and X lighter (in some specimens tergites V and IX also lighter). Scape of antenna without distolateral process (Fig. 41). Segment II of the maxillary palp without a distomedial concavity (Fig. 38). Segment II of labial palp with a broad apically rounded triangular distomedial projection (Fig. 39). Dorsal margin of femur (Fig. 40) with numerous setae proximally and rarely any distally; ventral margin with a few scattered setae. Dorsal margin of tibia with a few minute setae. Paraproct with approx. ten stout, pointed spines increasing in length towards apex (Fig. 44). **Male imago**: Genitalia with inner margin at the apex of segment I and base of segment II with a triangular well-marked expansion; segment III almost globular (Fig. 46).

#### Description.

*Larva*. Length: fully grown female: Body 6.2–8.0 mm, cerci 3.6–4.0 mm, terminal filament 2.4–2.8 mm. Fully grown male: Body 4.8–7.3 mm, cerci 3.3–3.6 mm, terminal filament 1.7–1.9 mm.

*Colouration* (Fig. 47): head almost uniformly medium brown, with darker, faint vermiform marking on vertex and frons, border of sclerites yellow. Prothorax medium brown with poorly marked yellowish pattern; mesothorax medium brown with a V-shaped yellow pattern; metathorax medium brown. Legs ecru except femora with a central brown spot and apex of femora, tibiae and tarsi brown. Tergites medium brown with small ecru spot except tergites I, V, VI, IX and X yellow, tergites V and VI generally with a dark M-shaped mark. Abdominal sternites ecru. Cerci ecru without dark stripe.

*Head*: scape of antenna without distolateral process (Fig. 41).

**Figures 34–39.**
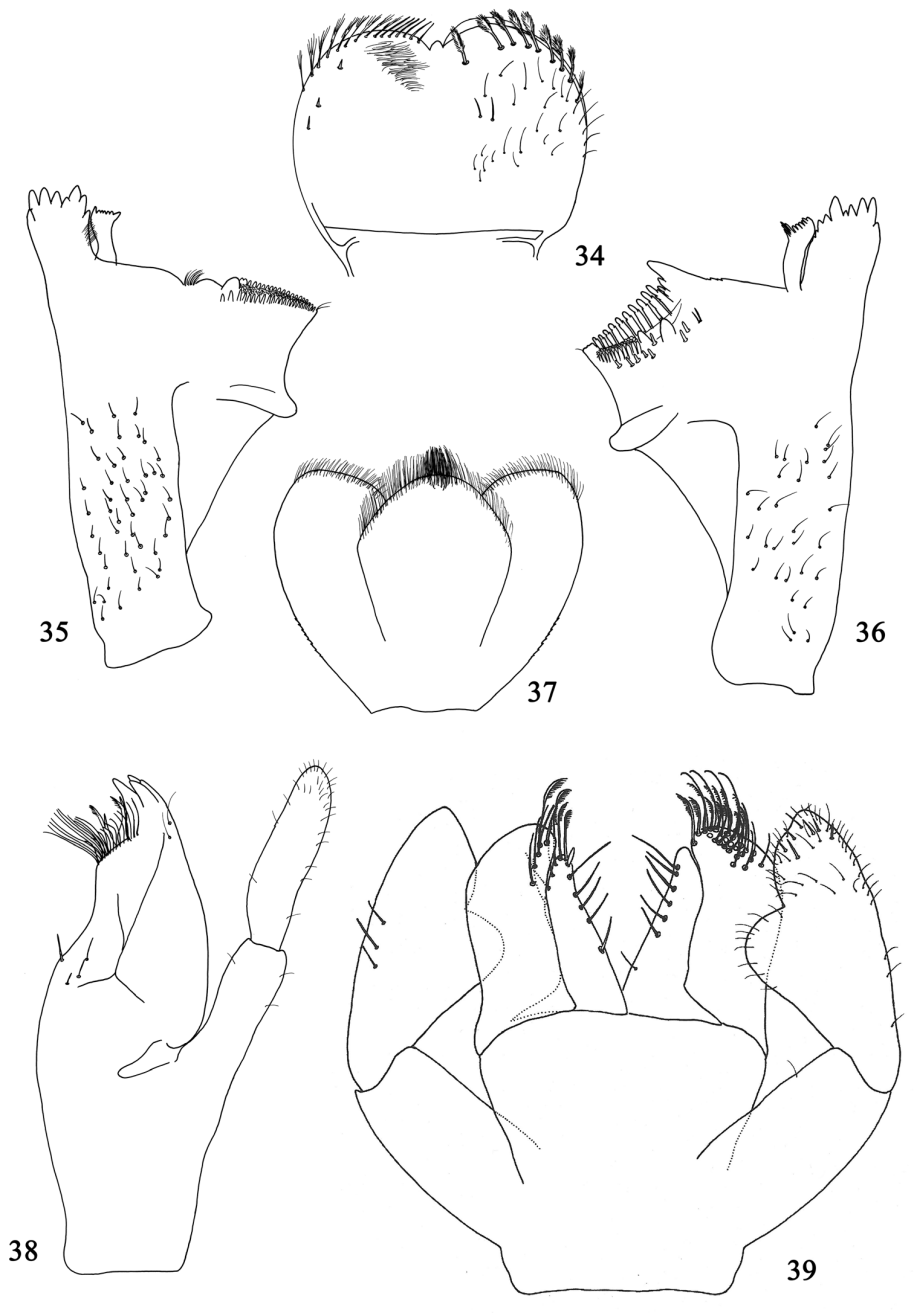
Larval structures of *Labiobaetis glaucus*: **34** labrum (left: ventral; right: dorsal) **35** right mandible **36** left mandible **37** hypopharynx **38** left maxilla **39** labium.

*Labrum* (Fig. 34) rounded, with a small anteromedial emargination, dorsally with one feathered submedian seta, and a distolateral row of approx. eight feathered setae; short, thin, simple setae scattered on dorsal surface of labrum; distal margin bordered with feathered setae.

*Right mandible* (Fig. 35): canine with two almost fused incisivi each with four denticles, outer denticle much shorter than others, inner margin of inner incisive with a row of very thin setae; stout prostheca apically with small rounded denticles; margin between prostheca and mola straight, smooth, without setae; tuft of setae at apex of mola reduced to two small setae.

*Left mandible* (Fig. 36): canine with two almost fused incisivi each with four denticles, outer denticle much shorter than others; stout prostheca apically with small denticles and a comb-shaped structure; margin between prostheca and mola straight, distally with crenulations; tuft of setae at apex of mola reduced to a single seta.

*Hypopharynx* as in Fig. 37.

*Maxilla* (Fig. 38) with a medioapical row of relatively short setae, basal end of row with approx. seven long setae; posterior side of lacinia mediobasally with a row of three medium-sized setae, a single small seta close to the medial margin of lacinia; palp 2-segmented, segment II without distomedial concavity.

*Labium* (Fig. 39) with glossae slightly shorter than paraglossae; glossae inner margin with two rows of approx. six long setae, apically with a few setae feathered on one side; paraglossae stout, apically rounded, with three rows of long setae, part of them feathered on one side; labial palp with segment I slender, shorter than segments II and III combined; segment II with a broad apically rounded triangular distomedial projection covered with thin setae, on posterior side with a row of three long setae; segment III subconical, inner margin apically slightly concave, with scattered short thin setae and a few stouter setae.

*Thorax*: hind wing pads present.

**Figures 40–44.**
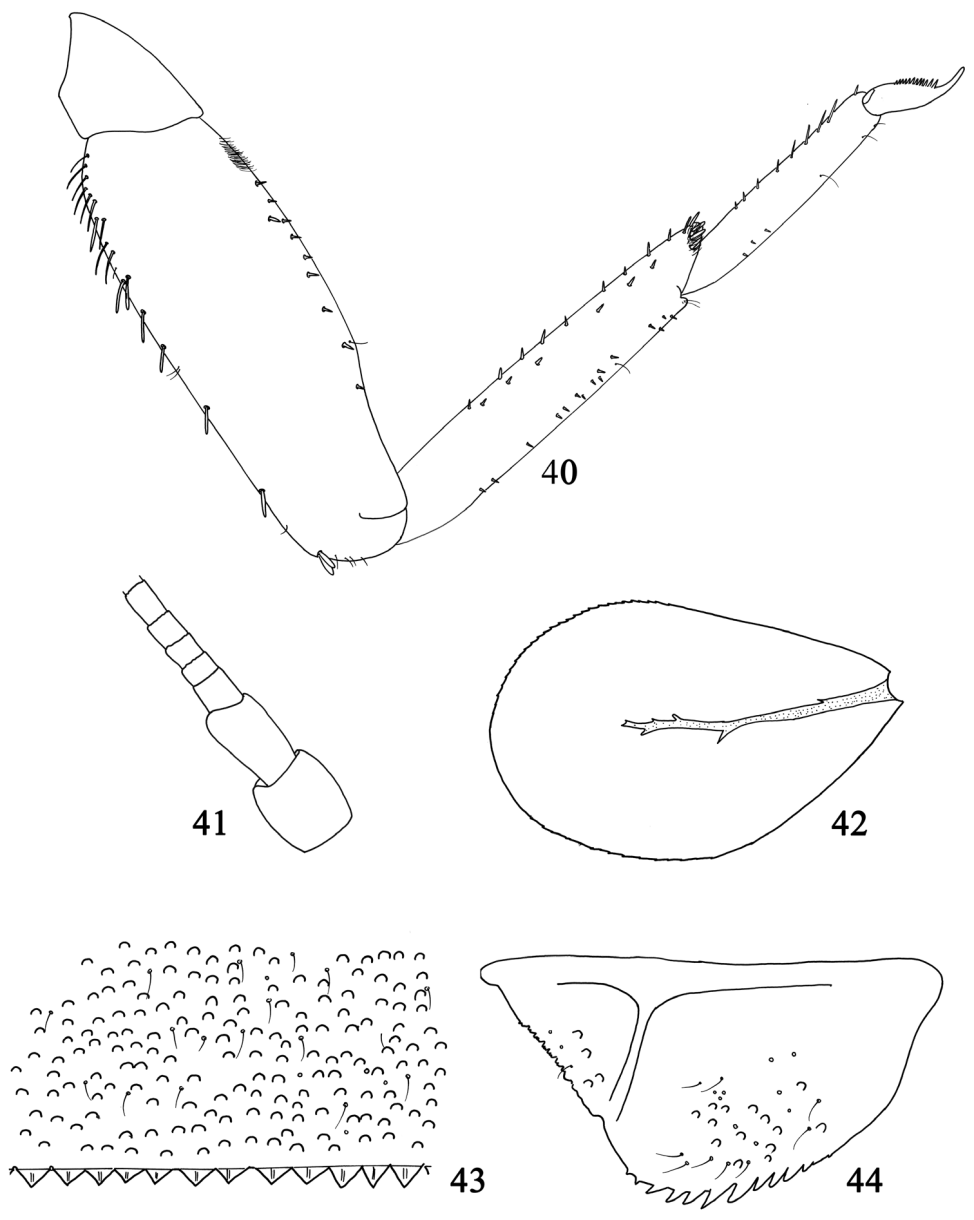
Larval structures of *Labiobaetis glaucus*: **40** foreleg **41** base of antenna **42** fourth gill **43** posterior margin of fourth abdominal tergite **44** paraproct.

*Legs* (Fig. 40): Forefemur dorsally with a row of medium-sized, apically rounded setae, numerous proximally and rare distally; apex with two short flattened setae; ventral margin with a well-developed villopore and scarce, short, stout setae. Foretibia dorsally with a row of scarce tiny, stout setae; ventrally with a few short setae, not longer apically, apex with a patch of numerous flattened short setae. Foretarsus almost bare dorsally; ventral margin with a row of pointed setae slightly increasing in length toward apex; tarsal claw with a single row of approx. twelve pointed teeth; subapical setae absent. Middle and hind legs similar to foreleg but with reduced setation.

*Abdomen*: tergites (Fig. 43) with numerous scale bases, with a few setae; posterior margin with short and broad triangular spination. Sternites with a few setae, without scales and scale bases; sternites I-VII with posterior margin smooth without spines, sternites VIII and IX with small triangular spines.

*Gills* present on abdominal segments I–VII, distally serrated, tracheation brown, poorly developed (Fig. 42).

*Paraproct* (Fig. 44) with scale bases and a few setae, margin with approx. ten stout, pointed spines increasing in length; posterolateral extension with a few scale bases, minute spines along the margin.

**Male imago.** *Length*. Body 4.4–4.5 mm; forewing 4.2–4.3 mm; hindwing 0.8 mm.

**Figures 45–47.**
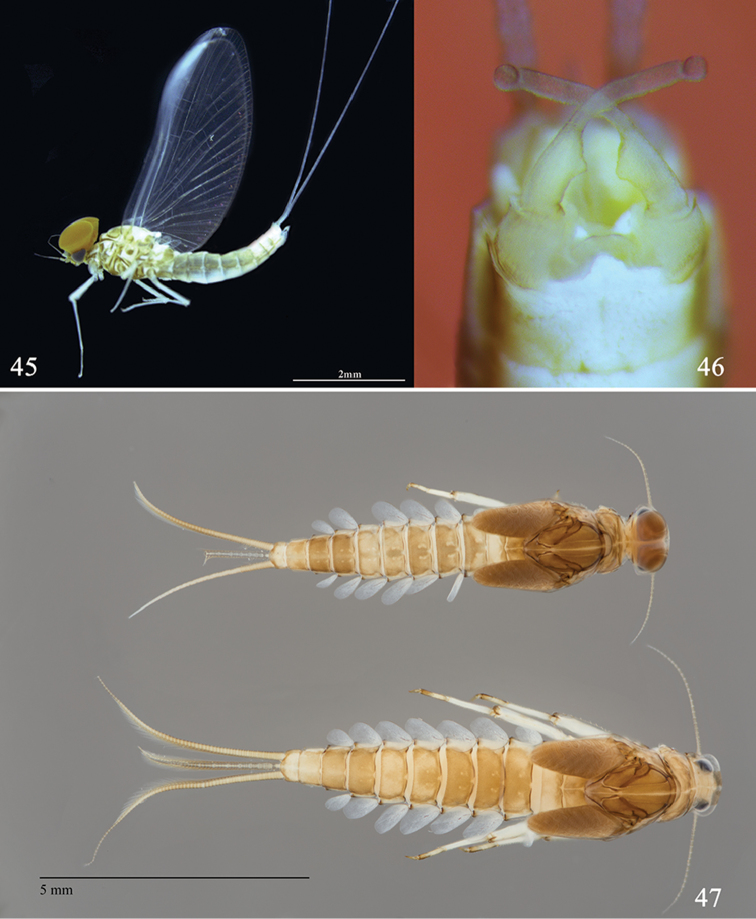
Larval and imaginal structures of *Labiobaetis glaucus*: **45** male imago (lateral view) **46** male genitalia **47** male and female larvae (dorsal view).

*Colouration*: head dark brown; antenna ecru. Facetted surface of turbinate eyes orange brown, shaft orange brown (Fig. 45). Thorax yellowish brown with margin of sclerites generally dark brown. Legs: yellowish without marks or pattern. Wings hyaline except costal and subcostal area apically white, with brown venation. Abdomen: tergites I to X light brown without mark or pattern. Sternites I and II light brown; sternites III to VII uniformly ecru without marks or pattern; sternites VIII and IX light brown. Cerci ecru. Genitalia (Fig. 46) ecru except inner margin of segment I medium brown. Forewing (Fig. 45): pterostigma with approx. four cross-veins not reaching subcostal vein; double intercalary veins shorter to almost equal to distance between corresponding main veins. Hindwing similar to Fig. 17 except two longitudinal veins reaching margin. Genitalia (Fig. 46): basal segment with inner margin not expanded apically; segment I and II almost completely fused; inner margin at the apex of segment I and base of segment II with a triangular well-marked expansion; segment III almost globular.

#### Distribution and habitat.

This species was collected in three different wadis at altitudes between 200 m and 750 m. Larvae occur in small streams, generally very shallow (a few centimeters to 20 cm) with moderate current. The substrate was a mix of sand, cobbles and rocks (Figs 50, 51, 52). This species was sympatric with the two other species of *Labiobaetis* and C. cf. soldani, but generally less abundant than other species.

**Figures 48–52.**
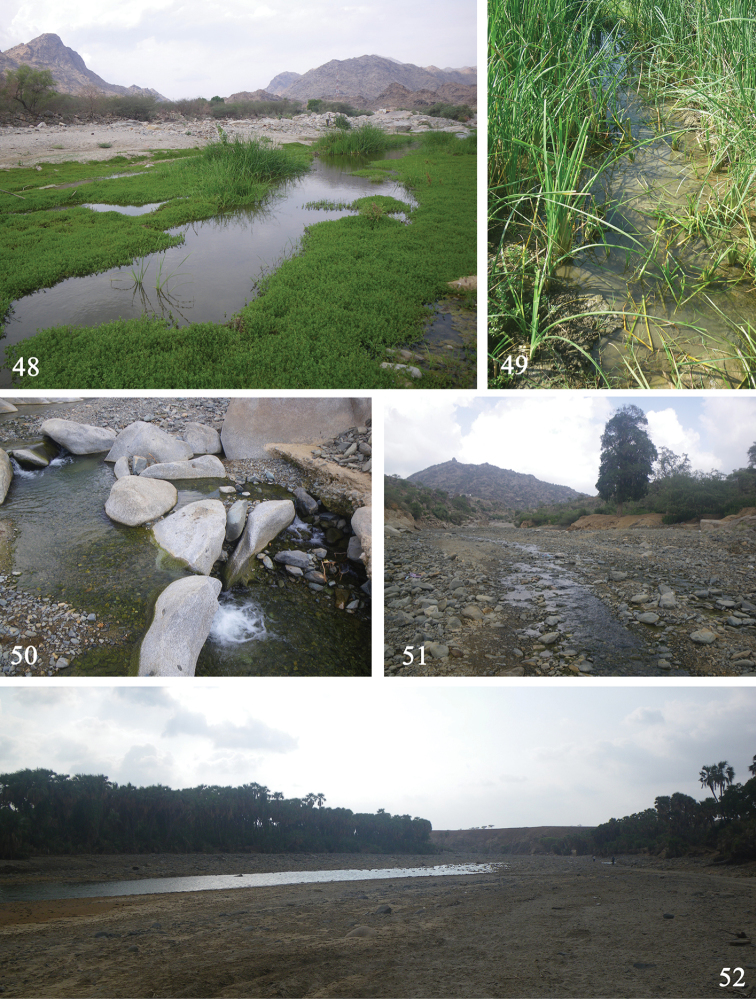
General aspects of *Labiobaetis* larval habitats: **48** AR32; Wadi Elarj, near Adam (Type locality of *Labiobaetis potamoticus*) **49** AR32; Wadi Elarj, near Adam: lateral channel with *Typha* sp. **50** AR43b; Wadi Shahadan: small waterfall **51** AR43a; Wadi Shahadan (type locality of *Labiobaetis alahmadii*) **52** AR44; Wadi Shahadan.

##### Molecular results

The mitochondrial reconstruction clearly recovers *L. potamoticus* and *L. alahmadii* as monophyletic clades (BS (Bootstrap support) of 83% and 100% respectively), with intraspecific K2P distances below 1% (Table 2). *Labiobaetis glaucus* is also highly supported as a monophyletic clade (BS of 100%), with the three populations (KSU, Mayotte, and South Africa) supported as monophyletic sister-clades (BS of 87%, 81% and 90% respectively). The sister group of *Labiobaetis potamoticus* is an undescribed species from South Africa; the distance between the two taxa is slightly higher than intraspecific distance (between 4.2 and 5.1%). *Labiobaetis potamoticus* possesses high distances to all the other species included in the study (16.2 to 25.5%). The relationships of *L. alahmadii* and *L. glaucus* with other species of Afrotropical and Palaearctic origins also are unclear and have no molecular support (Fig. 11). Both species are highly distant from any other taxa (Table 2).

**Figure 53.**
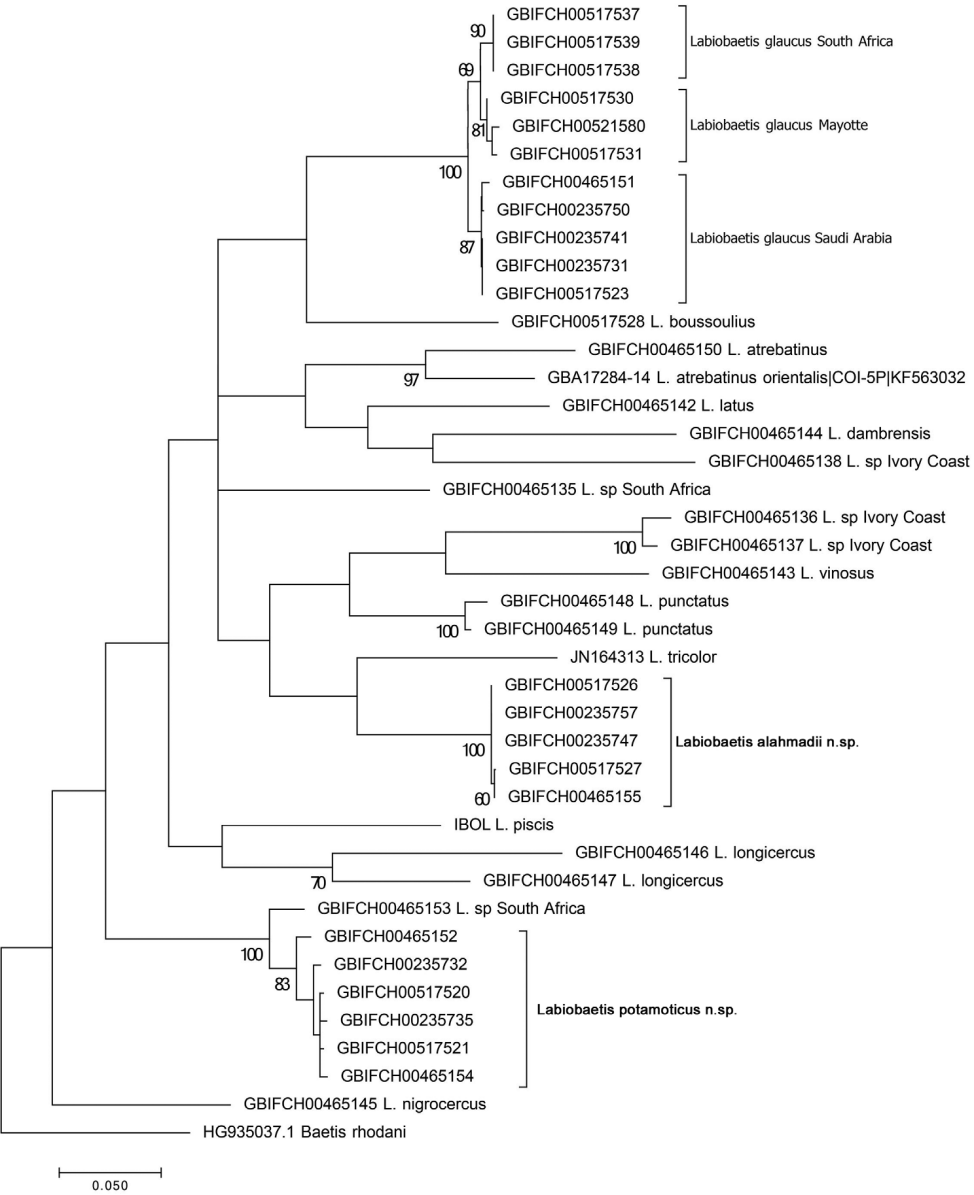
Maximum Likelihood (ML) consensus tree reconstructed for 40 haplotypes of *Labiobaetis* spp. Tree drawn to scale, branch lengths measured in number of substitutions per site, deeper nodes labelled above branches with Maximum Likelihood bootstrap support (>50%).

**Table 2.** Estimates of evolutionary divergence between major haplogroups of *Labiobaetis* species (using corrected p distances). In brackets are indicated the minimum and maximum distances.

	*Labiobaetis glaucus*	*Labiobaetis glaucus*	*Labiobaetis glaucus*	*Labiobaetis alahmadii*	*Labiobaetis potamoticus*
*Labiobaetis glaucus*	0 (0.0–0.004)				
*Labiobaetis glaucus*	0.02 (0.06–0.021)	0 (0.0–0.012)			
*Labiobaetis glaucus*	0.023 (0.021–0.025)	0.012 (0.012)	0		
*Labiobaetis alahmadii*	0.196 (0.194–0.199)	0.173 (0.173)	0.183 (0.183)	0	
*Labiobaetis potamoticus*	0.179 (0.173–0.183)	0.182 (0173–0.189)	0.168 (0.168)	0.168 0.162–0.173	0.003 (0.00–0.008)
*Labiobaetis* spp.	(0.178–0.254)	(0.178–0.250)	(0.173–0.255)	(0.153–0.293)	(0.042–0.256)

## Discussion

*Labiobaetis alahmadii* is morphologically similar to *L. glaucus* as well as with the Palaearctic species *L. cleopatrae* (Thomas & Soldán, 1989) and *L. balcanicus* and the Afrotropical species *L. boussoulius* (Gillies, 1993). The shape of the distolateral process of the second segment of the labial palp is of taxonomic importance to separate the different species: more elongated and curved in *L. potamoticus* (Fig. 8) and *L. balcanicus* whereas shorter and more rounded in *L. glaucus* (Fig. 39), *L. cleopatrae*, and *L. boussoulius*. *L. alahmadii* differs from these four species by the presence of short spatulate setae on the dorsal margin of the tibia and tarsi (Fig. 26) (Gillies 1993; Lugo-Ortiz et al. 2000; Müller-Liebenau and Soldán 1981; Thomas and Soldán 1989).

*Labiobaetis glaucus* is rather similar to *L. cleopatrae*. The two species differ by minor characters such as: the shape of the margin between prostheca and mola of the right and left mandibles (straight in *L. glaucus* (Fig. 35) and curved in *L. cleopatrae*), the shape of the apex of the segment II of the maxillary palp (simple in *L. glaucus* (Fig. 38) and slightly curved with a small apical protuberance in *L. cleopatrae*), distal margin of tergites (triangular and pointed simple spines in *L. glaucus* (Fig. 43) and double triangular slightly worn spines in *L. cleopatrae*), tarsal claw less curved and less stout in *L. glaucus* (Fig. 40) than in *L. cleopatrae.* These statements are based on the comparison of material of *L. glaucus* stored in the collection of the MZL with the original description of *L. cleopatrae.* According to the variability already noticed in *L. glaucus* and the known synonyms of the species (Lugo-Ortiz et al. 2000; Lugo-Ortiz and McCafferty 1997), we cannot exclude that *L. cleopatrae* is a possible junior synonym of *L. glaucus*.

*Labiobaetis potamoticus* and *L. piscis* Lugo-Ortiz & McCafferty, 1997 share several important synapomorphies: shape of the distomedial projection of segment II of the labial palp (Fig. 8); spines of the paraprocts (Fig. 15) and setation of the inner margin of the femur (Lugo-Ortiz and McCafferty 1997). These two species differ primarily by the shape of segment III of the labial palp (more slender and with the inner margin more concave in *L. piscis*) and the setation of the ventral margin of femora (villopore reduced but always present in *L. potamoticus*, absent in *L. piscis*; setae almost as long in ventral margin than dorsal margin in *L. piscis*, much shorter in *L. potamoticus*). *Labiobaetis potamoticus* and *L. piscis* show a peculiar pattern on the abdominal tergites (Fig. 19). A similar pattern is also present in *L. tripunctatus* Gillies, 1994 and *L. punctatus* Gattolliat, 2001 (Gattolliat 2001; Gillies 1994). *Labiobaetis tripunctatus* has no hindwings and only six pairs of gills (Gillies 1994), while *L. punctatus* is strictly endemic to Madagascar and clearly differs by the excavation of the maxillary palp segment II, the spination of the distal margin of tergites and the setation of the labrum (Gattolliat 2001). Genetically, *L. potamoticus* does not appear related to either *L. piscis* or *L. punctatus*. No sequences are available for *L. tripunctatus*. The sister group of *Labiobaetis potamoticus* is an undescribed species from South Africa. The two species are morphologically similar but also exhibit some differences especially in the femoral setation. This tends to confirm that the two sister taxa are closely related species but are not conspecific. The status of this undescribed species will be discussed in a revision of *Labiobaetis* from the Afrotropical Region (Kaltenbach, comm. pers.). *Labiobaetis potamoticus* was initially identified as *L. balcanicus* (Thomas and Sartori 1989). The two species share important characters (paraproct, antenna, labrum, mandibles, abdominal pattern), but also clearly differ by the shape of the spines of the distal margin of abdominal tergites (apically pointed in *L. potamoticus* (Fig. 14) whereas apically rounded in *L. balcanicus*) and the shape of the distomedial projection of the segment II of the labial palp (shorter and more slender in *L. potamoticus* (Fig. 8)).

The imagos of the different species of *Labiobaetis* are generally similar. The presence or absence of the hindwings and the shape of the genital plates are the main characters to separate the species (Gattolliat 2001). The male imago of *Labiobaetis potamoticus* cannot be separated from most other species of *Labiobaetis* with hindwings and broad, apically flat genital plates. *Labiobaetis glaucus* and *L. boussoulius* differ from most other species of the genus by the presence of a well-marked triangular expansion on the inner margin of the gonopods (Fig. 3 in Gillies 1993). A similar triangular expansion on the inner margin of the gonopods is present, but even more pronounced, in *L. tricolor* Tshernova, 1928 (fig. 111a in Müller-Liebenau 1969).

The discovery of *L. glaucus* in KSU is rather unexpected despite that this species is widely distributed in Afrotropical Region (South Africa, Angola, Kenya, Lesotho, Namibia, and Zimbabwe (de Moor et al. 2000; Lugo-Ortiz et al. 2000); Comoros islands (N. Mary unpublished data)). We examined specimens of populations from South Africa, Mayotte (Comoros Islands) and KSU. We found no clear morphological differences between them and they fully correspond to the original description and subsequent redescriptions (Agnew 1961; Lugo-Ortiz and McCafferty 1997; Lugo-Ortiz et al. 2000). As already mentioned (Lugo-Ortiz et al. 2000), segment II of the labial palp may have a more or less developed distomedial projection and the abdominal pattern may differ between populations. Genetically, populations from South Africa, Mayotte and KSU form a highly supported monophyletic clade. Genetic distances between the three populations are clearly of intraspecific range especially if we consider that intermediate populations from East and North-East Africa are not included in the analysis.

### Key to the larvae of *Labiobaetis* known from KSU

**Table:** 

1	Ventral margin of femora with abundant pointed setae (Fig. 10); labial palp with a moderately developed, relatively slender distolateral process (Fig. 8); margin of paraproct with abundant regular spines (Fig. 15); abdominal segments I-X brown with six ecru dots (Fig. 19)	***Labiobaetis potamoticus* sp. n.**
–	Ventral margin of femora with scarce short setae (Figs 25, 40); labial palp with a well-developed distolateral process (Figs 24, 39); margin of paraproct with 8–10 spines increasing in length (Figs 31, 44); abdominal segments I-X with different patterns	**2**
2	Dorsal margin of tibiae and tarsi with a complete row of spatulate setae (Fig. 26); mesothorax medium brown with a double V-shaped yellow pattern; tergites I-VIII medium brown with two broad ecru spot; tergites IX and X yellow (Figs 32, 33)	***Labiobaetis alahmadii* sp. n.**
–	Dorsal margin of tibiae and tarsi with scattered minute pointed setae; mesothorax (Fig. 40) uniformly brown (Fig. 47); tergites II-IV and VII-VIII medium brown with small ecru spot, other tergites yellow	***Labiobaetis glaucus***

## Supplementary Material

XML Treatment for Labiobaetis potamoticus

XML Treatment for Labiobaetis alahmadii

XML Treatment for Labiobaetis glaucus
